# Set membership experimental design for biological systems

**DOI:** 10.1186/1752-0509-6-21

**Published:** 2012-03-21

**Authors:** Skylar W Marvel, Cranos M Williams

**Affiliations:** 1 Department of Electrical and Computer Engineering, North Carolina State University, Raleigh, NC 27695, USA

## Abstract

**Background:**

Experimental design approaches for biological systems are needed to help conserve the limited resources that are allocated for performing experiments. The assumptions used when assigning probability density functions to characterize uncertainty in biological systems are unwarranted when only a small number of measurements can be obtained. In these situations, the uncertainty in biological systems is more appropriately characterized in a bounded-error context. Additionally, effort must be made to improve the connection between modelers and experimentalists by relating design metrics to biologically relevant information. Bounded-error experimental design approaches that can assess the impact of additional measurements on model uncertainty are needed to identify the most appropriate balance between the collection of data and the availability of resources.

**Results:**

In this work we develop a bounded-error experimental design framework for nonlinear continuous-time systems when few data measurements are available. This approach leverages many of the recent advances in bounded-error parameter and state estimation methods that use interval analysis to generate parameter sets and state bounds consistent with uncertain data measurements. We devise a novel approach using set-based uncertainty propagation to estimate measurement ranges at candidate time points. We then use these estimated measurements at the candidate time points to evaluate which candidate measurements furthest reduce model uncertainty. A method for quickly combining multiple candidate time points is presented and allows for determining the effect of adding multiple measurements. Biologically relevant metrics are developed and used to predict when new data measurements should be acquired, which system components should be measured and how many additional measurements should be obtained.

**Conclusions:**

The practicability of our approach is illustrated with a case study. This study shows that our approach is able to 1) identify candidate measurement time points that maximize information corresponding to biologically relevant metrics and 2) determine the number at which additional measurements begin to provide insignificant information. This framework can be used to balance the availability of resources with the addition of one or more measurement time points to improve the predictability of resulting models.

## Background

Costly materials, limited resources, and lengthy experiments are constraints that hinder our ability to acquire quantifiable measurements from biological systems. Experimental design approaches are computational techniques for extracting the most useful information from experiments yet to be performed [1]. These techniques are needed for the study of biological systems to conserve the limited resources that are allocated for performing experiments. Application of these techniques to biological systems has introduced novel mathematical algorithms and models to life sciences, while also requiring the development of new mathematical theories and programming tools [2]. An important aspect of experimental design for biological systems is model calibration, which requires the estimation of parameters such as kinetic and diffusivity constants [3]. The development of accurate biological models is constrained by the financial costs and time required to perform biological experiments, often leading to a collection of sparse datasets with which to estimate the parameters of proposed model structures. Experimental design provides a method to yield the best estimates from data given the limitations in data collection, component observability and limited system excitability.

The development and application of experimental design has a rich history spread across a wide range of fields. An excellent review article by Pronzato has condensed the underlying concepts behind the most widely used techniques of experimental design for nonparametric and parametric models [1]. The reader is referred to the review article and the works cited therein for a thorough understanding of statistical methods for experimental design.

Typically, parameter estimation problems begin by claiming that observations $\hat{y}$ are perturbed from ideal model outputs **g**(**x**, θ*) by an error *ε*, such that

(1) $${\hat{y}}_{i}=g\left(x_{i},\theta^{*}\right)+\varepsilon_{i},i=1,\cdots \;,k,$$

where **x**_*i *_are the model states at ***k ***different times or experimental conditions, θ* are the true parameter values and the errors, ε*_i_*, are statistically independent with zero mean and variance $E\left(\varepsilon_{i}^{2}\right)=\sigma^{2}\left(x_{i}\right)$. It is assumed that the errors can be defined by probability density functions, often assumed to be independent and identically distributed Gaussian random variables with zero mean and variance σ^2 ^for mathematical convenience. The unknown parameter vector can then be determined by the maximum likelihood estimate ${\hat{\theta }}_{ML}^{k}$. As *k *→ ∞ the difference between ${\hat{\theta }}_{ML}^{k}$ and θ* can be described by a normal distribution with zero mean and covariance matrix, Σ, which is bounded from below by the inverse of the Fisher Information Matrix (FIM) according to the Cramér-Rao inequality [1].

Experimental design aims to maximize information, or minimize uncertainty, about unknown model parameters by exploring experimental configurations such as the sampling times where new measurements should be acquired, the desired number of measurements to add, which system components should be measured, etc. The criteria used to evaluate the information of a design are derived from scalar functions of the FIM [1]. A-optimal design, for example, minimizes trace(FIM^-1^), or equivalently minimizes of the sum of squared lengths of the axes of asymptotic confidence ellipsoids for ***θ***. E-optimality refers to designs where the longest axis of asymptotic confidence ellipsoids for ***θ ***is minimized, which is equivalent to maximizing the minimum eigenvalue of the FIM. D-optimal design maximizes det(FIM) and corresponds to minimizing the volume of asymptotic confidence ellipsoids for ***θ***.

Although there is a large body of work dedicated to experimental design using statistical methods [1], several problems arise when using these approaches for the modeling of biological systems [4]. Kreutz and Timmer state several of the difficulties in using experimental design for biological systems: i) models are often large and the number of measurements is very limited, ii) relative noise levels of 10% or more are standard for biochemical data, iii) little prior knowledge exists. These considerations make it difficult to correctly characterize the distribution of uncertainty in the model, which is the primary pillar upon which FIM approaches for experimental design are based. Even if the correct distribution is obtained, accurate parameter estimations using the FIM are usually valid only when a large number of data points are available, which is not often the case for biological systems [5]. Rather, the finite range of values that system component concentrations can take on at a given time more appropriately characterizes the uncertainty in biological systems. This bound can be inferred based on the experimental technology, the characteristics of limited replicates, and/or first principles knowledge. Therefore, a set membership framework is more appropriate for the development of experimental design for many biological systems, where the error is bounded with no other hypothesis given regarding its distribution [6].

A key aspect of experimental design for bounded-error models is how to characterize the set of parameter values that are consistent with all data measurements. Initial methods for constructing this set use conservative bounding approaches based on ellipsoids to characterize the parameter sets. More precise parameter set estimations can be obtained using interval analysis [7,8], *but these interval techniques have not previously been applied to experimental design approaches*. Apart from the method used to bound the parameter set, proper experimental design metrics are important because they provide a logical link between physical resources and mathematical constructs. Traditional experimental design criteria for bounded-error models minimize the volume of parameter sets that are consistent with the data [6,9-11]. However, the information provided by this metric may not be useful to a biologist. Other metrics that are related directly to the uncertainty of specific parameters or the effects on unmeasurable model states may be of more interest. Such biologically relevant information can be obtained from simple criteria functions previously not used in experimental design for bounded-error models. Set membership experimental design methods have recently regained attention. Hasenauer et al. have developed a set-based experimental design method using semidefinite programming with V-optimality as the only design metric [12]. The expected information content from additional measurements is determined using a Monte-Carlo approach to simulate different parameters, input sequences and measurement errors. While this method demonstrates the usefulness of bounded-error techniques, there is a lack of connection between the design metric and biological interpretation. Additionally, the use of a Monte-Carlo approach to simulate the effect of additional measurements requires a large number of simulations and can be very time consuming. Bounded approaches, such as the one we outline in this paper, allow for the impact of uncertainty to be assessed without needing to perform Monte-Carlo simulations.

In this work, we develop an experimental design framework that utilizes interval analysis to generate the set of parameters and state bounds consistent with all data measurements. This approach leverages many of the recent advances in bounded-error parameter and state estimation methods [7,8], including increased accuracy through the use of interval analysis instead of bounded ellipsoids, as the base of our experimental design framework. Our novel framework uses parameter and state estimations based on initial data measurements, which may provide data for only a subset of the model states, to estimate measurement bounds at candidate time points of interest to the experimenter (times when measurements have not been taken). We then use these estimated measurements at the candidate time points to evaluate which candidate measurements furthest reduce model uncertainty. We propose a method for combining candidate time points to determine the effect of adding multiple measurements. We present biologically relevant design metrics to evaluate candidate designs in order to address issues associated with making a better connection between modelers and experimentalists. These contributions comprise a bounded-error experimental design framework that can be applied to nonlinear continuous-time systems when few data measurements are available. This framework can be used to balance the availability of resources with the addition of one or more measurement points to improve the predictability of resulting models.

## Methods

In this section, we define a specific experimental design problem and outline how our framework is used to determine the number of additional measurements that are warranted and at what time points these measurements should be taken. The relevant interval arithmetic algorithms for parameter and state estimation used throughout this process are briefly presented. We show how to select a set of candidate time points based on the estimated state bounds of a proposed model given initial data measurements and provide a method to estimate the corresponding candidate measurement bounds. Techniques for determining the effect of adding multiple candidate time points on parameter and state estimations are discussed. We define several biologically relevant metrics, which are scalar functions of the parameter and state estimations after incorporating estimated candidate time point measurements. These metrics can convey information such as the activity of specific enzyme kinetic parameters or bounding values for the estimation of unmeasured component concentrations.

### Problem statement

Consider the following ordinary differential equation (ODE) model of a biological system:

(2) $$\begin{array}{l} \dot{x}=f(x(t),\theta )\\ y=g(x(t),\theta ), \end{array}$$

Where **x **∈ ℝ*^n ^*is an n-dimensional vector of component concentrations, **y **∈ ℝ*^m ^*is an m-dimensional vector of measurements, and ***θ ***∈ ℝ*^p ^*are the *p *model parameters. An initial set of bounded data measurements has been obtained at *k *different times:

$Y:=\left\{{\hat{y}}_{i}|{\underline{y}}_{i}\leq y\left(t_{i}\right)\leq {ȳ}_{i};i=1,\cdots \;,k\right\}$, where *i *is the index corresponding to time *t_i _*and ${\underline{y}}_{i}$ and ${\bar{y}}_{i}$ are the lower and upper measurement bounds, respectively. The problem under study is to determine at what time points to collect new data measurements for minimizing or maximizing specific parameter and/or state information metrics.

**Figure 1:**
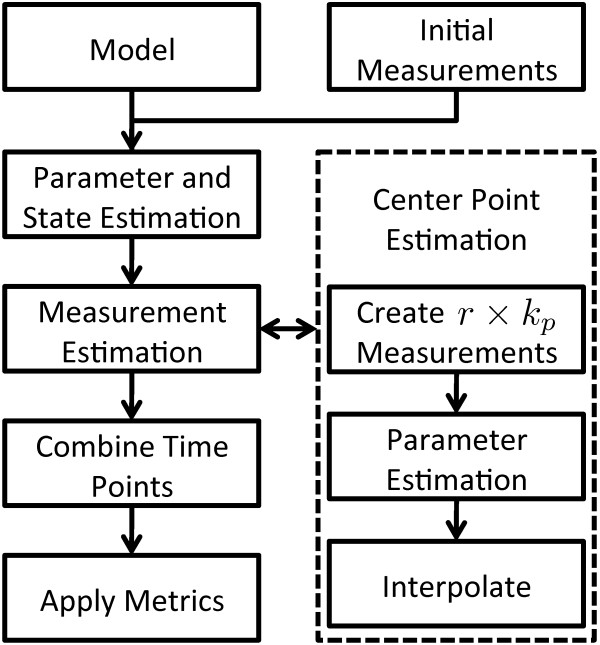
**Experimental design method**. This figure outlines a block diagram of the experimental design approach. The process is outlined in four major steps (shown on the left). A novel approach for estimating measurement bounds at candidate time points is implemented.

We use the method outlined in the left half of Figure 1 to solve this problem using a set membership approach by applying biologically relevant information metrics to evaluate candidate time points. First, we perform bounded parameter estimation using the initial bounded-error measurements. Estimated state bounds are then generated from the resulting parameter space. Second, a set of candidate time points is selected from locations where relatively large uncertainties exist in the estimated model states. We propose a novel approach to estimate the measurement bounds at candidate time points using a set-based approach that incorporates the initial bounded-error measurements adjacent to each candidate time point. Third, we perform bounded parameter and state estimations that incorporate the candidate measurements to predict the possible effects of adding a measurement at the corresponding time point. We also assess the impact of adding multiple measurements on the resulting estimates. As a proof of concept, we compare the performance of our estimated measurements and true measurements at each candidate time point, assessing the ability of estimated measurements to predict which candidate time point most reduces a given uncertainty metric. We assess this for single and combinations of candidate time points. We also use our estimated measurements at each candidate time point to identify the 'point of diminishing return' where additional measurements no longer provide additional information, leading to no further decrease in estimate uncertainty.

### Bounded estimation

These methods use interval analysis to computationally guarantee a valid bounded-error solution to the system of ODEs by employing interval box enclosures that bound the states during integration steps. Methods have been introduced in the literature to address overestimation due to wrapping effect [13-15] and to help reduce the computational burden for estimating parameters of complex, higher dimensional models [16], which are typical for biological processes.

### Uncertainty propagation

Interval analysis is a form of guaranteed computing and can be used to generate solutions to ODEs through the use of interval boxes and inclusion functions [17]. Consider the model function **g**, which maps a state interval box [**x**] to the corresponding image in the data space **g**([**x**]). Here the interval box [**x**] represents the Cartesian product of *n *scalar intervals [**x**] = [*x*_1_] × [*x*_2_] × ⋯ × [*x_n_*], where [*x_i_*] represent the interval $\underset{}{x_{i}}\leq x_{i}\leq {\bar{x}}_{i}$. A non-minimal inclusion function, , is a non-unique mapping from state space to data space and contains the smallest interval box that encloses the image **g**([**x**]).

Computing the solution of ODEs for *t*_0 _≤ *t *≤ *t_N _*with time step *h *is done using Taylor expansions [17-19]. This method involves an inflation step where the bounds of the remainder term for the *k*^th^-order Taylor expansion of the model ODEs are inflated by 1 ± α. Evaluation of the Taylor expansion is performed using the Extended Mean Value (EMV) algorithm proposed by Rihm [19] using mean value forms [20] and matrix preconditioning. Whenever the EMV algorithm generates state values, [**x**], at a time where data measurements, $\left[\hat{y}\right]$, are available, Set Inversion Via Interval Analysis (SIVIA) [21] is used to compare the two.

### Set inversion

SIVIA is able to determine solution sets for unknown quantities **u **from a functional relationship **q(u) **= [**y**]. An *a priori *search space for **u **is recursively explored using SIVIA to determine a guaranteed enclosure of the solution space. The resulting solution space is comprised of feasible and indeterminate boxes. These boxes, **[u]**, are determined from the following relations: if **q([u]) **⊆ **[y] **then **[u] **is *feasible*; if **q([u]) **∩ **[y] = ***ϕ *then **[u] **is *unfeasible*; else **[u] **is *indeterminate*. Indeterminate boxes are bisected and tested again until its widest dimension reaches a user specified threshold ε > 0.

### Parameter and state estimation

The methods presented in this paper leverage the works of Jaulin for state estimation [7] and Raïssi et al. for parameter estimation [8]. Parameter estimation combines the EMV and SIVIA algorithms to systematically evaluate candidate boxes in the parameter space. Our framework uses these two algorithms to build our set-based experimental design approach. We perform parameter estimation by evaluating hypercubes in the partitioned parameter space to identify if each hypercube or box produces trajectories that are consistent with the measurements obtained from the system. A parameter box that produces trajectories that are inconsistent with any data measurement is classified as unfeasible and discarded. Any parameter box that produces a trajectory determined by SIVIA to be completely contained within all data measurements is labeled as feasible. All other parameter boxes are labeled as indeterminate. These indeterminate boxes are bisected and retained for further evaluation. We apply this bisection process recursively to any indeterminate box where the width of the widest dimension is larger than a user-defined length, ε > 0. We implemented the augmented estimation method presented by Marvel and Williams to enable its application for systems where few data measurements are available (Marvel S, Williams C: Set Membership and Parameter Estimation for Nonlinear Differential Equations Using Discrete Measurements, Submitted). We estimate bounds on the resulting component concentrations consistent with the data measurements by executing the EMV algorithm using the parameter boxes classified as feasible and indeterminate. This state estimation will not only produce bounds between data measurements of measured states, but also provide bounds for unmeasurable states. We parallelized this method using the Message Passing Interface (MPI) protocol to distribute the boxes across multiple processors to effectively distribute computations across available processing resources [22].

### Estimating candidate measurements

The measurements at a given time are characterized by an upper and lower bound such that $\underset{}{y}\leq y\leq \bar{y}$. Mathematically, this measurement can be defined by three values: 1) the time *t_j _*at which the measurement was observed, 2) the center point **C***_j_*, and 3) its range **R***_j _*such that |**C***_j _*- **y **(*t_j_*)| ≤ **R***_j_***/**2. We estimate the center points and ranges of candidate measurements using the bounds of adjacent data measurements and the estimated bounds on component concentration trajectories generated by the EMV algorithm. Once estimated, each candidate measurement is added to the original *k *data measurements to assess the impact of the additional measurement information on our ability to estimate the parameters and unmeasured states. We describe below how *t_j_*, **C***_j_*, and **R***_j _*are estimated for candidate measurements.

To simplify notation in this subsection, we assume that one or more of the states can be directly measured (**y **= **x**). This will allow for direct comparison between estimated state bounds and measurement values. This is a common assumption made for biological systems [7,8]. In a more general case, comparisons would require use of the inclusion function  to compare G(x) and **y **via SIVIA.

### Time point and range estimation

For a given state, time points for candidate measurements are chosen by first identifying all times *t *between measurements at *t*_*i *_and *t*_*i*+1_, whose estimated range (generated by the EMV algorithm) is greater than or equal to both of the measurement uncertainties at times *t*_*i *_or *t*_*i*+1_. This presents a worst case scenario because we are selecting candidate time points with the most possible uncertainty. Alternative time points can be selected based on practical experimental limitations or first principles knowledge. The set of time intervals, , for a corresponding state can be written as

(3) $$\begin{array}{c} T:=\left\{t\left|\bar{x}\left(t\right)-\underset{-}{x}\left(t\right)\geq \text{max}\right)\left[\bar{x}\left(t^{-}\right)-\underset{}{x}\left(t^{-}\right),\right)\right)\\ \left(\left(\bar{x}\left(t^{+}\right)-\underset{-}{x}\left(t^{+}\right)\right]\right\} \end{array}$$

where $t^{-}=\max_{t_{i}}\left(t_{i}<t\right)$ and $t^{+}=\min_{t_{i}}\left(t_{i}>t\right)$. Selecting candidate time points from the intervals in  is an empirical task. For example, a total of *k_p _*candidate time points could be selected from within the interval set  based on a collection of physically feasible time slots where measurements can be observed. The set of candidate time points is denoted as $T:=\left\{t_{j};j=1,\cdots ,k_{p}\right\}$, with T⊂T. The corresponding candidate time point ranges are determined based on a conservative premise that uses uncertainty information contained in adjacent measurements. Here, we set the range of candidate measurements to be

(4) $$R_{j}=\text{max}\left[\left(\bar{x}\left(\overset{-}{\underset{j}{t}}\right)-\underset{-}{x}\left(\left(\overset{-}{\underset{j}{t}}\right)\right),\left(\bar{x}\left(\overset{+}{\underset{j}{t}}\right)\right)-\underset{-}{x}\left(\overset{+}{\underset{j}{t}}\right)\right)\right],$$

where $t_{j}^{-}=\max_{t_{i}}\left(t_{i}<t_{j}\right)$ and $t_{j}^{+}=\min_{t_{i}}\left(t_{i}>t_{j}\right)$. The amount of information available for determining appropriate range values is limited when no probabilistic assumptions are imposed on the uncertainty. Here, *R_j _*is a relatively conservative estimate that assumes the uncertainty of the system at a new candidate time point is not less than that of data measurements taken near the same time.

### Center point selection

Center point estimation is conservatively implemented to reduce the chance of erroneously eliminating valid kinetic parameters and component concentrations. We introduce a novel approach for estimating the corresponding center point of each candidate time point. This approach estimates the position of the center point *C_j _*that maximizes the resulting parameter estimate volume at given time *t_j _*and range *R_j_*. The three main steps in this process are shown in the right half of Figure 1. First, *r *measurements are simulated at each $t_{j}\in T$ by shifting *R_j _*from the lower bound to the upper bound on the estimated state bounds. For example, if *r *= 3 and the estimate of state *x *at time *t*_4 _is bounded between the range [3,6] with *R*_4 _= 1.5, the resulting shifted candidate measurements at time *t*_4 _would have bounds [3,4.5], [3.75,5.25] and [4.5,6]. Second, bounded parameter estimation is performed for each of the *r *shifted candidate measurements for each of the *k_p _*candidate time points. Curve fits for each set of *r *parameter volumes are used to determine the center point, *C_j_*, that maximizes the parameter volume for each candidate time point *t_j_*. This allows us to fully construct conservative measurement estimations for candidate time point *t_j _*using *C_j _*and *R_j_*.

### Combining measurements

The ability to investigate the effects of adding multiple measurements is often desirable when designing biological experiments. Employing a brute-force method for assessing the impact of all combinations of candidate measurements at $t_{1},t_{2},\cdots \;,t_{k_{p}}$ on estimated kinetic parameters and component concentrations is a large computational burden. A brute-force approach for exploring combinations of up to *k_c _*candidate time points would require computing parameter and state estimations for $\sum_{m=2}^{k_{c}}\left(\begin{array}{c} k_{p}\\ m \end{array}\right)$ measurement sets. This is potentially problematic even for systems of low dimension and few unknown parameters. We hypothesize that there exists a level of independence among candidate time points that can be exploited to speed up our ability to evaluate the impact of multiple measurements on parameter uncertainty.

The estimated parameter space for a combination of candidate time points, $P_{c}$, can be obtained by intersecting the parameter estimates of the individual time points, e.g. $P_{c}=P_{1}\cap P_{4}\cap P_{9}$. Computing the intersection of a set of non identical boxes is not an obvious task. We developed a simple approach for forming the union of sets of nonuniformed shaped boxes by bisecting the larger feasible boxes until all boxes have widths less than ∈. This approach allows boxes to be directly compared between estimated parameter sets. More sophisticated approaches can be applied that will preserve the largest possible feasible boxes during the intersection process. The estimated state bounds resulting from this combination of additional candidate measurements, **x**_c_, is then determined using the resulting intersected parameter boxes.

### Metrics

Scalar functions of the estimated parameter set and state bounds are used as metrics to predict the impact of adding measurements at candidate times *t_j _*on kinetic parameter and component concentration estimates. The metrics in this section can be conceptually related to traditional stochastic experimental design criteria functions (e.g. D-optimality, E-optimality, A-optimality [23]). However, the computation of these bounded-error metrics require no assumptions about underlying stochastic distributions of the model parameters or system states and relate directly to the physical components of the system. Thus, the biological interpretation of the bounded-error metrics is straightforward since they can be directly related to biological concepts instead of the mathematical construct of the FIM.

### Parameter volume

We will evaluate the parameter volume as a means to compare our new metrics to traditional V- and D-optimality design criteria [6]. This metric will predict the candidate time points that minimize the volume of the estimated parameter space. The parameter volume, $P_{V}$, can easily be calculated by summing the volumes of the interval boxes,

(5) $$P_{V}=\underset{i}{\sum }\overset{p}{\underset{j=1}{\prod }}\Delta p_{i}^{j},$$

where $\Delta p_{i}^{j}$ is the width of the j^*th *^dimension of the *i^th ^*parameter box. A drawback of this metric is the inability to detect large uncertainties in potentially important parameters if they are masked by less important but well known parameters. To combat this, the parameters could be weighted based on biological importance, giving more weight to parameter dimensions deemed important by the experimenter.

### Parameter bounds

This metric can be customized for predicting candidate time points based on the uncertainty of a single parameter or a subset of parameters. Single parameter values are compared using the width of the uncertainty for the parameter of interest, e.g. $P_{p_{i}}={\bar{p}}_{i}-\underline{p_{i}}$. Multiple parameters are compared using the Euclidean norm to produce a scalar value from the widths of uncertainty for the selected parameters, e.g. $P_{||p_{i},p_{j}||2}={\left[{\left({\bar{p}}_{i}-\underline{p_{i}}\right)}^{2}+{\left({\bar{p}}_{j}-\underline{p_{j}}\right)}^{2}\right]}^{1/2}$.

### State bounds

This metric utilizes estimated state bound information and allows the experimenter to see how estimated ranges of unmeasured states are affected by additional measurements. This may be of interest when constraining the range of state values is more important than parameter information. Also, the information provided by this metric is biologically meaningful because it provides a predicted limit on state values such as component concentrations. This metric is computed similarly to the parameter bounds metric but with the parameter uncertainties replaced by the maximum ranges of estimated states. Other custom metrics are also possible; for example, designing a metric to select the time points that minimizes the maximum value of a specific state.

## Results and discussion

In this section, the proposed experimental design method is applied to an example problem. We evaluate our set-based experimental design approach by performing a proof of concept on a model that has been used in the literature to evaluate several other set-based approaches [7,8,14,15]. Our problem set-up is more stringent than the approach outlined in [8] because we assume only a small set of data measurements from a single state is available as opposed to assuming data measurements are available at every time step. We use our approach to predict at what time additional measurements should be made in order to identify the candidate measurements that maximize information corresponding to previously defined metrics and to determine the number at which additional measurements begin to provide insignificant information.

### Problem setup

The model under examinations is the Lotka-Volterra predator prey model, which is a canonical biological ODE model [24] and serves as a key model for testing algorithms in this field. This is a two-state model and is described by the following differential equations:

(6) $$\begin{array}{c} {ẋ}_{1}=x_{1}\left(p_{1}-p_{2}x_{2}\right)\\ {ẋ}_{2}=-x_{2}\left(p_{3}-p_{4}x_{1}\right), \end{array}$$

where *x*_1 _is the prey population, *x*_2 _is the predator population, *p*_1 _is the prey birth rate, *p*_2 _is the decrease in prey population due to encounters with predators, *p*_3 _is the predator death rate, and *p*_4 _is the increase in predator population due to encounters with prey. This model was used by Raïssi et al. to demonstrate their bounded parameter estimation algorithm when data measurements of the prey population are available for all N = 1,400 time points between *t*_0 _= 0 and *t_N _*= 7.

Initial data measurements were simulated by first generating model state values using exact inputs to the EMV algorithm and then adding uncertainty. The underlying state values, x*, were generated using the same initial state values, model parameters and EMV algorithm settings as those used by Raïssi et al.: x_1_(*t*_0_) = 50, *x*_2_(*t*_0_) = 50, *p*_1 _= 1, *p*_2 _= 0.01, *p*_3 _= 1, *p*_4 _= 0.02, α =0.005, *h *= 0.005 and *k *= 4 for 0 ≤ t ≤ 7. Three initial data measurements were generated by adding random uncertainty to the true state values in order to create interval bounds at discrete time points, far fewer than the *N *≥ 1,000 measurement time points used in prior literature involving this model [8,15]. The seconds state, *x*_2_, was assumed to be unmeasurable while for the first state, *x*_1_, measurements were generated by adding error intervals as follows: ${\hat{x}}_{1}\left(t_{i}\right)=x_{1}^{*}\left(t_{i}\right)+\varepsilon_{i}$, where *ε_i _*= [-8.2190, 13.6065], [-11.3067, 14.9691] and [-7.6254, 10.5414] at *t_i _*= {2, 4 and 6}, respectively.

The assigned task is to determine at what times additional measurements would provide useful information with regards to the previously defined metrics and how many measurements would be beneficial. It was assumed that the initial conditions of both populations and parameters *p*_1 _and *p*_3 _were exactly known. We first wish to estimate the set of parameters *p*_2 _and *p*_4_, along with the range of the unmeasured state *x*_2 _for 0 ≤ *t *≤ 7, that are consistent with the uncertain measurements of *x*_1_.

### Initial parameter and state estimation

**Figure 2:**
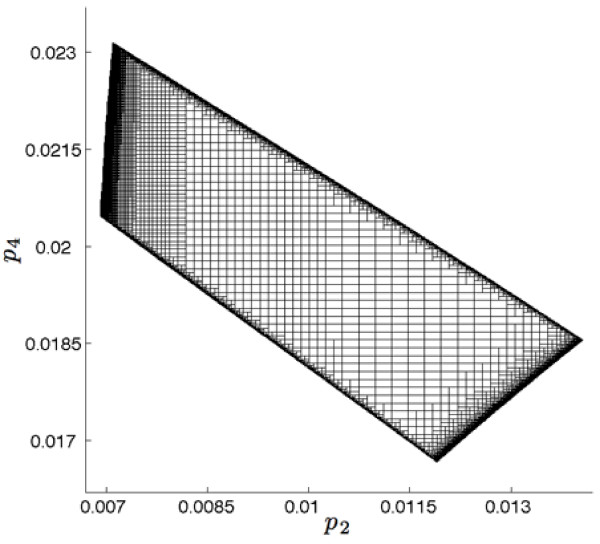
**Initial parameter estimate**. This figure shows the feasible and infeasible boxes in the parameter space that result from the SIVIA algorithm. No distinction between feasible and infeasible is shown.

**Figure 3:**
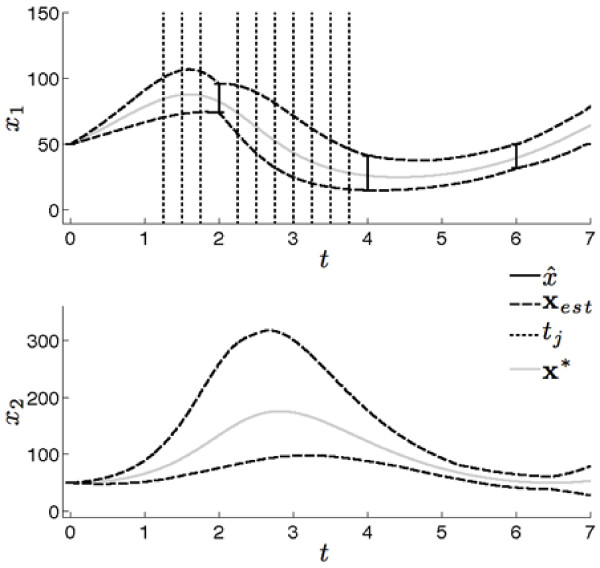
**Initial estimated state bounds**. The true state values resulting from *x*_1_(*t*_0_) = *x*_2 _(*t*_0_) = 50 and *p *= [1, 0.01, 1, 0.02] are shown in grey (x*). The initial measurement set is shown as uncertainty bounds in x_1 _at *t *= 2, 4, and 6 $\left({\hat{x}}_{i}\right)$. Recall that measurements are only available for x_1_. The dashed lines show the results of the uncertainty propagation of the estimated parameter boxes in Figure 2 (**x**_*est*_). The dotted lines show the positions of candidate times points (*t_j_*).

Bounded estimates of parameters *p*_2 _and *p*_4 _and states *x*_1 _and *x*_2 _were calculated using the initial measurements $Y:=\left\{{\hat{x}}_{1}\left(t_{i}\right);i=1,2,3\right\}$ Parameter estimation was performed assuming an *a priori *search area of [-1, 1] for both *p*_2 _and *p*_4 _and indeterminate boxes were bisected until a minimum box width of ε = 10^-5 ^was obtained. This resulted in the generation of ~20 k indeterminate and feasible boxes shown in Figure 2 where no distinction is made between the two box types. Each box was then used in the EMV algorithm to produce the estimated state bounds, **x**_*est*_, shown in Figure 3 where x* are the grey waveforms, ${\hat{x}}_{i}$ are the intervals and **x**_*est *_are the black dashed waveforms.

### Estimating candidate measurements

The initial data measurements were compared to the estimated state bounds for *x*_1 _to generate the interval set  from which the candidate time points will be selected. Here, *k_p _*= 10 candidate time points were chosen from within , namely T={1.25,1.5,1.75,2.25,2.5,2.75,3,3.25,3.5,3.75} as indicated in Figure 3. The corresponding values of *R_j _*and *C_j _*were estimated for each candidate time point *t_j _*using the approach described above. The ranges *R_j _*were shifted along the estimated state bounds for each corresponding *t_j _*using *r *= 15 steps, where *r *was determined empirically to obtain curve fits with large R^2 ^values. Bounded parameter estimations were performed for the *k_p _*× *r *= 150 shifted candidate measurements. The estimated parameter volumes were fit to quadratic curves with resulting R^2 ^values greater than 0.99. We were then able to identify an estimate of the center point that maximized this curve.

### Combining time points

**Figure 4:**
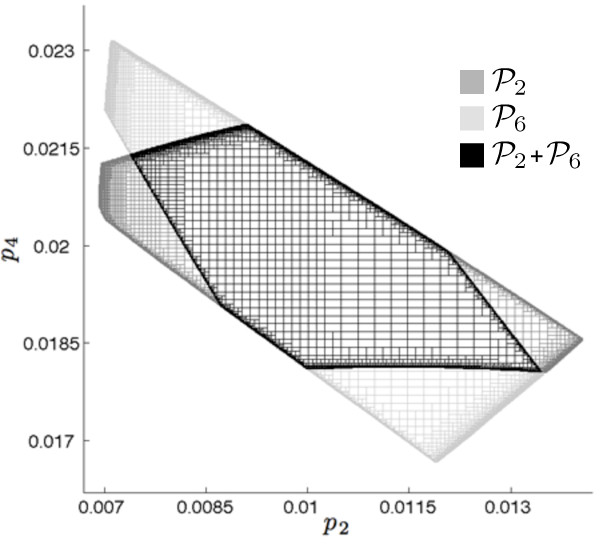
**Parameter space intersection**. This figure shows the estimated parameter uncertainty assuming a candidate measurement at *t*_2 _was added ($P_{2}$, dark grey boxes) and the estimated parameter uncertainty assuming a candidate measurement at *t*_6 _was added ($P_{6}$, light grey boxes). The black boxes show the brute-force combination of $P_{2}$ and $P_{6}$. It is clear that the intersection of $P_{6}$ and $P_{6}$ is equivalent to the brute-force combination.

We were able to establish independence between candidate time points by showing that the brute-force estimates using all possible permutations and the intersected parameter sets cover identical parameter regions. The brute-force combinations and the intersections of parameter sets for all combinations of two candidate time points were compared and found to produce both the same parameter volumes and parameter bounds with a tolerance of 10^-12^. Parameter intersections were then computed for combinations of up to *k_c _*= 5 candidate time points. An example parameter intersection is shown in Figure 4 where the parameter estimates of *t_2 _*= 1.5 and *t_6 _*= 2.75 were combined. The parameter box colors correspond as follows: dark grey for $P_{2}$, which corresponds to *t*_2_, light grey for $P_{6}$, which corresponds to *t*_6_, and black for the brute-force combination which is used to depict the intersected parameter space.

**Figure 5:**
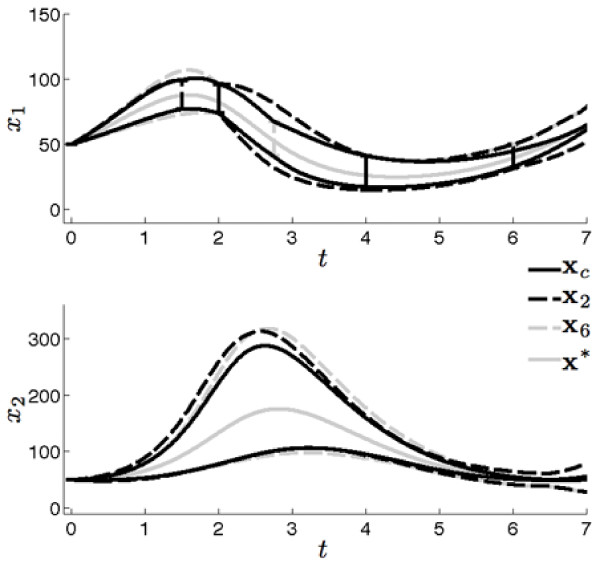
**Combination of estimated state bounds**. This figure shows the estimated state bounds assuming a candidate measurement at *t*_2 _was added (**x**_2_, dashed black lines) and the estimated state bounds assuming a candidate measurement at *t*_6 _was added (**x**_6_, dashed grey lines). The estimated state bounds for the combined candidate measurements, **x**_*c*_, are the black lines, while the underlying true state values, x*, are the solid grey lines.

Estimates of state bounds were computed from the intersected parameter sets. An example estimate of state bounds is shown in Figure 5 for the parameter intersection of *t*_2 _and *t*_6_. The underlying state values x* are the solid grey waveforms, the combined estimated state bounds **x**_c _are the solid black waveforms and the estimated state bounds **x**_2 _and **x**_6_, corresponding to the results obtained from adding candidate measurements at *t*_2 _and *t*_6_, respectively, are the dashed black and dashed grey waveforms, respectively. The decrease in uncertainty for state *x*_2 _during 1 ≤ *t *4 is caused by the removal of the non-overlapping parameter regions.

### Applying metrics

We tested whether the estimated candidate measurements generated by our algorithm could effectively be used to predict where the most appropriate measurements should be placed to reduce model uncertainty. With this in mind, we generated a set of true measurements at each candidate time point using the underlying state values, x*, as the true center points, C*. We consider estimates obtained from measurements characterized by the true center points to be *ground truth*, corresponding to the best estimate of the measurement at a specific candidate time point. The metric results for estimates using the true center points C* are used as a reference and compared to the results obtained when using our estimated center points *C_j _*.

### Parameter information

**Figure 6:**
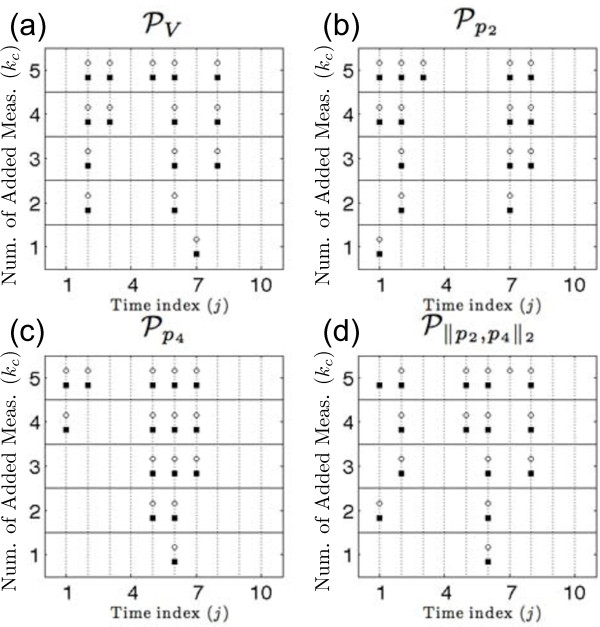
**Best candidate measurements for parameter metrics**. This figure illustrates the location of the best candidate measurement (x-axis) given the number of potential measurements that can be added (y-axis) for a given metric. The index value of predicted time points are represented by solid squares for C* and open circles for *C_j_*. (a) Candidate time point locations to best reduce parameter volume ($P_{V}$). (b) Candidate time point locations to best reduce uncertainty in $p_{2}\left(P_{p_{2}}\right)$. (c) Candidate time point locations to best reduce uncertainty in $p_{4}\left(P_{p_{4}}\right)$. (d) Candidate time point locations to best reduce uncertainty in both *p*_2 _and *p*_4 _$\left(P_{||p_{2},p_{4}|{|}^{2}}\right)$.

The prediction of the best time point locations, given the set of candidate measurements, for several parameter metrics are shown in Figure 6 when using center points C* (solid squares) and *C_j _*(open circles). This figure shows the best candidate measurement time point locations relative to the index of *t_j _*for the parameter volume metric, $P_{V}$, individual unknown parameter bounds, $P_{p_{2}}$ and $P_{p_{4}}$, and combination of parameter bounds, $P_{||p_{2},p_{4}|{|}^{2}}$ Consider the design approach when there are only enough resources for a single additional measurement. Selecting a design to minimize the uncertainty of parameter *p*_2 _(Figure 6b) would suggest placing a measurement at time *t*_1 _= 1.25. However, to minimize the uncertainty of parameter *p*_4 _a measurement at time *t*_6 _= 2.25 would be more beneficial. If there are resources available for three additional measurements they would best be placed at times *t_2 _*= 1.5, *t*_6 _= 2.75, and *t*_8 _= 3.25 to obtain additional information on both unknown parameters. We emphasize the established consistency between the best candidate time points selected based on C* and the best candidate time points selected based on our estimate *C_j_*. The only inconsistent prediction between center points C* and *C_j _*occurs when applying the $P_{||p_{2},p_{4}|{|}^{2}}$ metric for a combination of *k_c _*= 5 time points, which results in a single time point difference.

**Figure 7:**
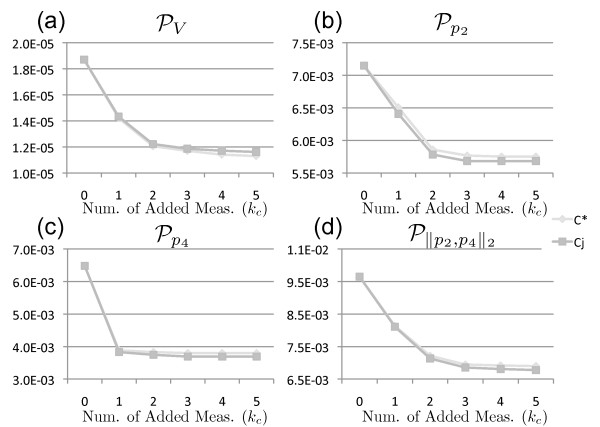
**Parameter metric values**. Plots of parameter metric values vs number of additional measurements. These plots demonstrate the decrease in parameter uncertainty with additional measurements. The point of diminishing return is indicated by the elbow of the curve for the respective metric. This shows that additional measurements will no longer decrease uncertainty associated with that metric.

The point at which additional measurements will not provide any additional information about the system can be predicted by observing the metric values for combinations of time points. This is especially beneficial for conserving resources that would otherwise be spent on experiments that yield no new information. The values of the four parameter metrics are shown in Figure 7 as functions of the number of additional measurements. Using this information, an experimental designer could determine the desired number of additional measurements to collect without wasting resources. Consider selecting a set of measurements to reduce uncertainty for parameter *p*_4_. Estimating the impact of adding multiple measurements leads to the clear conclusion that a single additional measurement is all that is required. Similarly, reducing the uncertainty of the consistent parameter set volume may require 2 or 3 additional measurements. These metric value curves can be combined with cost functions to determine a design that efficiently utilizes experimental resources.

### State information

**Figure 8:**
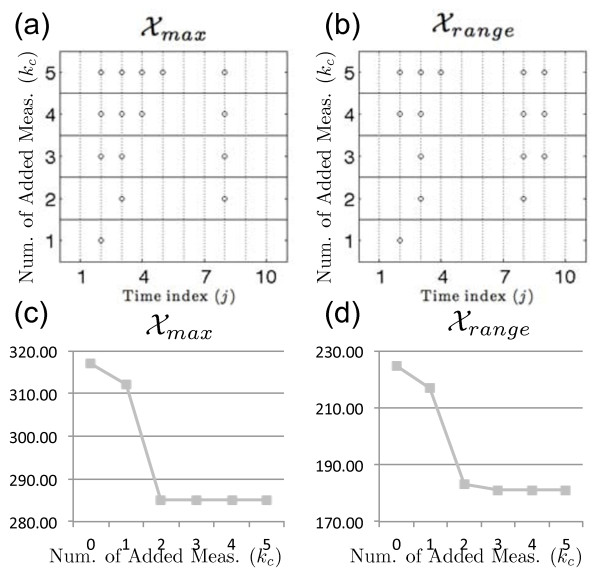
**Best candidate measurements and metric values for state metrics**. Best candidate measurements for state metrics and corresponding metric values. Candidate time point locations are indicated by open circles for center points *C_j_*. These metrics are used to determine the impact of additional measurements from state *x*_1 _on the estimated state bounds of *x*_2_.

Two metrics were applied to the unmeasured state, *x*_2_, to determine how its uncertainty is impacted when candidate measurements are applied to state *x*_1 _using center points *C_j_*. The first metric, $X_{max}$, was used to select candidate time points that would minimize the overall maximum value of *x*_2_. The second metric, $X_{range}$, determines which candidate measurements will minimize the maximum uncertainty of *x*_2 _over the simulation time 0 ≤ *t* ≤ 7. The best time point locations and corresponding metric values are presented in Figure 8. Candidate measurement locations are fairly similar for the two metrics with $X_{max}$ slightly favoring candidate measurements located at earlier time points. A dramatic increase in information can be seen for both metrics when increasing from a single additional measurement to a combination of two measurements (Figure 8c-d). Little knowledge is gained when adding three or more measurements when compared to that gained from two additional measurements.

### Comparison with FIM D-optimality

Scalar metrics of the Fisher Information Matrix (FIM) are often used to perform experimental design for many conventional problems [1,23,25]. We compared the results of our set-based experimental design approach to results obtained using the D-optimality metric of the FIM. We did this to show how statistical assumptions that are often made to calculate the FIM could potentially impact the results when performing experimental design for biological processes. As stated previously, the number of measurements obtained for biological systems is very limited [4]. These data points are used to impose unwarranted statistics on the uncertainty, which are then used to calculate the FIM. Consider the scenario often encountered when quantifying biological systems where resources are available for only four replicates of a given experiment, i.e. only four data points are generated for a given sample time *t_i_*. The sets {74, 75, 80, 95}_1_, {74, 80, 89, 95}_2 _and {74, 89, 94, 95}_3 _show three likely data sets containing four data points from experimental replicates for sample time *t_i_*. All sets show data in the interval range 74 to 95. The small sample size of each set, however, implies that meaningful statistics of the uncertainty are difficult to obtain. In fact, each set has distinctively different means, with *μ*_1_, *μ*_2_, and *μ*_3 _corresponding to 81, 84.5, and 88, respectively. Given that the use of the FIM inherently assumes the use of Gaussian distributions [26], we use our results below to assess how these imposed Gaussian distributions, with their potentially different means, impact the decisions associated with experimental design.

**Figure 9:**
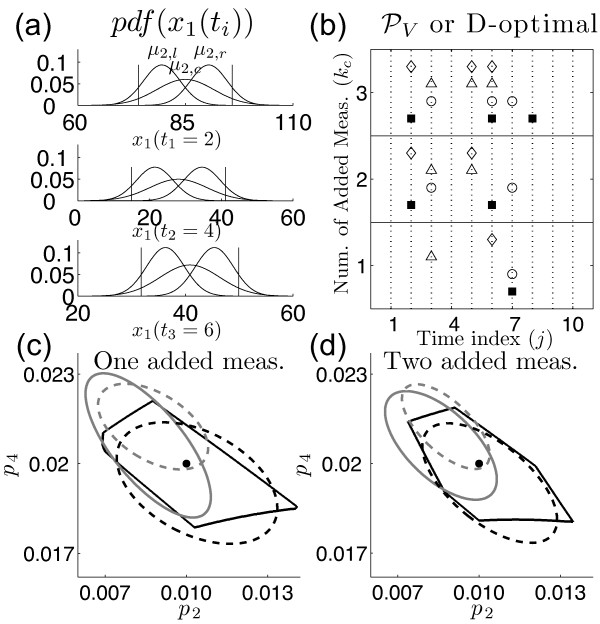
**Comparison with D-optimal design**. This figure compares our set-based $P_{V}$ experimental design to FIM D-optimal design when using measurements that are characterized by Gaussian distributions. (a) Figure illustrating different possible Gaussian distributions for each of the three original measurement sample times (*t*_1 _= 2, *t*_2 _= 4 and *t*_3 _= 6). The three distributions for each sample time are characterized by left shifted, center shifted, and right shifted means. (b) Time index of predicted time points given the number of additional measurements that can be made. The figure shows a comparison of time point selection for the following: solid squares--set-based method, circle $-\theta_{ML}^{\left\{c_{2},c_{4},c_{6}\right\}}$, triangle $-\theta_{ML}^{\left\{c_{2},l_{4},l_{6}\right\}}$ and diamond $-\theta_{ML}^{\left\{l_{2},l_{4},l_{6}\right\}}$. (c-d) Parameter estimations after adding one or two additional data measurements, respectively; black dot--θ*, solid black line--set-based method, dashed black line $-\theta_{ML}^{\left\{c_{2},c_{4},c_{6}\right\}}$ solid grey line $-\theta_{ML}^{\left\{c_{2},l_{4},l_{6}\right\}}$, dashed grey line $-\theta_{ML}^{\left\{l_{2},l_{4},l_{6}\right\}}$. These results show the importance of accurate distribution characterization when designing experiments using the FIM.

We looked at three possible Gaussian distributions for each of the original measurement times, *t_i _*= {2, 4, and 6}, that could result from having small numbers of data samples (Figure 9a). Each distribution is characterized by its mean, $\mu_{t_{i},s}$, and variance, $\sigma_{t_{i},s}^{2}$. The variable *t_i _*represents one of the original measurement time points and the variable *s *corresponds to the position of the distribution, i.e. *s *= l for shifted to the left, *s = c *for shifted to the center, and *s = r *for shifted to the right. All variances, $\sigma_{t_{i},s}^{2}$, were calculated such that the distribution had a probability of 0.9 over the original interval uncertainty range. This ensures that each distribution, even though they have different means, has the same probability of producing population values over the uncertainty interval range.

We calculated the Maximum Likelihood (ML) estimate of the parameters [27] for the nine possible combinations of these distributions given the three initial measurement time points, *t_i _*= {2, 4, and 6},

(7) $${\hat{\theta }}_{M\text{L}}^{\left\{s_{2},s_{4},s_{6}\right\}}=\underset{\theta }{\text{min}}\underset{t_{i}}{\sum }\frac{1}{\sigma_{t_{i},s}^{2}}\left({\left(x_{1}\left(t_{i},\theta \right)-\mu_{t_{i},s}\right)}^{2}\right),$$

where $S_{t_{i}}$ corresponds to the distribution type at time *t_i_*. For example ${\hat{\theta }}_{ML}^{\left\{l_{2},r_{4},c_{6}\right\}}$ is the ML estimation resulting from using the left shifted distribution at time *t*_1 _= 2, the right shifted distribution at time *t*_2 _= 4, and the center distribution at time *t*_3 _= 6. We computed the sensitivity matrix, *S*, using the method outlined in [28] by solving the ODE

(8) Ṡ=JS+A,

in combination with (6). Here, the (i, j)^th ^element of these variables are *S*_*i,j *_= ∂*x_i_*/∂*θ_j_, J_i,j _*= ∂*f_i _*/∂*x_j _*and *A*_*i,j *_= ∂*f_i _*/∂*θ_j_*. The FIM was then calculated as

(9) $$FIM={\left(\underset{t_{i}\in I}{\sum }\frac{1}{\sigma_{t_{i},s}^{2}}\frac{\partial x_{1}\left(t_{i}\right)}{\partial \theta^{T}}\frac{\partial x_{1}\left(t_{i}\right)}{\partial \theta }\right|}_{{\hat{\theta }}_{ML}^{\left\{S_{2},S_{4},S_{6}\right\}}},$$

where *I *is the set of original measurement time points {2, 4 and 6} in addition to the subset of candidate time points, *t_j_*, being evaluated, e.g. *ℐ *= {2,2.75,3.5,4,6} where 2.75 and 3.5 would be the two candidate time points being evaluated. The variances at candidate time points were characterized in a way that was consistent with our set-based approach. The variance $\sigma_{t_{j},s}^{2}$ for candidate time point *t_j _*was selected as the larger of the two variances of the adjacent initial measurements.

We computed D-optimal designs for the 9 distribution combinations and compared the selected candidate time points with our set-based method. The prediction of the best time point locations, given the set of candidate measurements, for our method (solid squares) and several D-optimal designs $(\text{circ}1\text{e}-\theta_{ML}^{\left\{c_{2},c_{4},c_{6}\right\}}$, triangle $-\theta_{ML}^{\left\{c_{2},l_{4},l_{6}\right\}}$ and diamond $-\theta_{ML}^{\left\{l_{2},l_{4},l_{6}\right\}}$) are shown in Figure 9b. The fluctuations in time point selection show that D-optimality is sensitive to our ability to correctly characterize the distributions of the initial data measurements, i.e. correctly characterizing the mean. Figure 9c and 9d show the corresponding parameter estimations for our method (solid black line) and the 95% confidence ellipsoids of D-optimal designs (dashed black line $-\theta_{ML}^{\left\{c_{2},c_{4},c_{6}\right\}}$, solid grey line $-\theta_{ML}^{\left\{c_{2},l_{4},l_{6}\right\}}$, dashed grey line $-\theta_{ML}^{\left\{l_{2},l_{2},l_{2}\right\}}$ after adding one and two measurements, respectively. The true parameter values are indicated with a point at (0.01,0.02). We are able to conclude based on these results that the selection of the time points for additional measurements, along with the assessment of the parameter uncertainty, changes depending on the characterization of the probability associated with the measurement uncertainty. Mischaracterization of the probability distribution is particularly possible when working with few data points, as is the case when modeling biological systems. This emphasizes the utility of our set-based experimental design approach. We also note that Figure 9c and 9d show that the resulting parameter uncertainty calculated using the FIM approach can result in an under or over estimation of the parameter range, depending on the characterization of the measurement uncertainty. This could be an important limitation in FIM experimental design approaches if one was interested in metrics related to absolute values of the parameter uncertainty (maximum value) instead of the relative change (minimum volume).

## Conclusions

Developing accurate models is crucial for understanding, predicting and ultimately controlling biological processes. The limitation of costly resources and lengthy experiments associated with the study of biological systems promotes an experimental design approach for model development. Stochastic experimental design methods rely on correctly characterizing the distribution of uncertainty in the model, often requiring a large number of data measurements. This requirement is difficult to fulfill for many biological systems and alternative set-based experimental design approaches are more appropriate in these situations. In addition to the method used to characterize uncertainty, biological interpretations of experimental design metrics are important because they provide a logical link between physical resources and mathematical constructs.

We have developed a novel experimental design framework using bounded-error methods and biologically relevant design metrics to select desirable time point locations where additional measurements will be collected for the purpose of improving resource allocation for biological experiments. Our method propagates the uncertainty resulting from a small collection of data measurements, which may contain information for only a subset of the model states, through time to estimate parameter and state bounds for a given system model. We used these bounded-error results to estimate candidate measurement time points, center points and ranges. We proposed a method for combining candidate time points and present several biologically meaningful design metrics.

Measurement estimation is an important component of this method. We used a set-based approach to estimate measurements at time points where no information was available. We were able to estimate measurement bounds at candidate time points by combining information from the initial data measurement bounds with the estimated state bounds generated by the EMV algorithm. Our method resulted in a good estimate when compared to true measurements for the purpose of identifying where additional measurements should take place. The granularity of candidate time points can be made as fine as desirable at the cost of additional computation time. The computational expense to search all possible time points may make identifying globally optimal time point locations impractical using this method. However, the accuracy of when measurements are collected during biological experiments is often on the order of minutes, hours or days and locally optimal time points from an experimentally feasible set of time points is often sufficient.

The ability to estimate the effects of adding measurements at multiple time points is often desirable. A brute force method to explore all combinations of time points is computationally expensive. However, we found that the parameter estimation for a combination of time points can be directly obtained by intersecting the individual estimated parameter spaces. Estimated state bounds can then be determined using the intersected parameter space. The experimenter can determine when additional measurements will provide little or no additional information by exploring the effects of adding multiple measurements and will not needlessly spend limited resources on experiments that yield no additional information.

The framework presented here can be used to predict at what time additional measurements should be made to maximize information based on biologically relevant metrics and to determine the number at which additional measurements being to provide insignificant information. Problems of this sort are often faced by biologists when modeling biological processes. Selecting an appropriate metric is made more straightforward by associating it with biologically relevant information. For example, the uncertainty of a parameter may be associated with specific characteristics of an engineered enzyme, while the limitations on the uncertainty of estimated state bounds can provide critical bounds on unmeasured component concentrations, allowing systems to maintain chemical and physiological phenotypes.

## Competing interests

The authors declare that they have no competing interests.

## Authors' contributions

SM designed the study and prepared the manuscript. CW participated in the design and in revising the draft. Both authors read and approved the final manuscript.
